# Identification of the plant mitochondrial *OrfX* protein: A mass spectrometry approach

**DOI:** 10.1002/1873-3468.70417

**Published:** 2026-07-20

**Authors:** Matthias Döring, Jan de Jonge, Hans‐Peter Braun, Nils Rugen

**Affiliations:** ^1^ Institute of Plant Genetics Leibniz Universität Hannover Germany

**Keywords:** complex III, complexome profiling, mTatC, orfx, plant mitochondria, respiratory chain, RNA editing, Tat translocase

## Abstract

The mitochondrial genome of plants contains an open reading frame, *orfx*, which encodes a protein classified as very rare and which has so far escaped mass spectrometric detection. The protein resembles the c‐subunit of bacterial twin‐arginine‐motif‐dependent protein translocases (TatC). Using native prefractionation of mitochondrial protein complexes from Arabidopsis and trapped‐ion mobility spectrometry (TIMS) time‐of‐flight mass spectrometry, we report the identification of three peptides of the *orfx* protein. Our experimental approach was used to trace the native forms of the protein and show that it is present in protein complexes in the size range of 450–530 kDa, which also contain TatB. We suggest that mitochondrial TatBC complexes in plants may bind to late assembly intermediates of respiratory chain complex III.

## Graphical abstract

The mitochondrial genome of plants contains an open reading frame, *orfx*, which encodes a rare protein that has so far escaped mass spectrometric detection. The protein resembles the c‐subunit of bacterial twin‐arginine‐motif‐dependent protein translocases (TatC). Using native prefractionation of mitochondrial protein complexes from Arabidopsis and highly sensitive mass spectrometry, we provide clear evidence for the expression of this putative pseudogene.

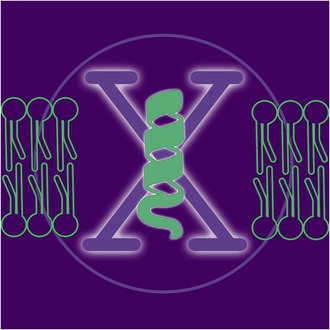

## Abbreviations

BN‐PAGE, blue‐native polyacrylamide gel electrophoresis

dda‐PASEF, Data‐dependent acquisition Parallel Accumulation Serial Fragmentation

dia‐PASEF, Data‐independent acquisition Parallel Accumulation Serial Fragmentation

MS, mass spectrometry

mTatB, mitochondrial subunit B of bacterial twin‐arginine‐motif‐dependent protein translocases

mTatC, mitochondrial subunit C of bacterial twin‐arginine‐motif‐dependent protein translocases

SSR, sequence‐specific retention

TIMS, trapped‐ion mobility spectrometry

TOF, time‐of‐flight

With the beginning of the characterization of plant mitochondrial genomes, an open reading frame (ORF) was discovered that encodes a protein which initially could not be functionally assigned. The ORF was named *orfx* [1, 2]. The gene is conserved in the mitochondrial genomes of all investigated plants; it is transcribed and transcripts are edited at several defined positions. It was later uncovered that the putative protein encoded by *orfx* resembles bacterial and chloroplast TatC, a subunit of the twin‐arginine translocation (Tat) protein export system [3, 4]. Proteins that are transported across membranes via the Tat translocase are characterized by a signal sequence containing a twin‐arginine motif.

The Tat translocase is present in the cytoplasmic membranes of many bacteria and archaebacteria and also in the thylakoid membranes of chloroplasts [5]. In contrast to the centrally important Sec protein secretion system, the Tat translocase is capable of transporting fully folded proteins.

In *E. coli*, the translocase consists of the three membrane proteins TatA, TatB, and TatC. While TatC is a polytopic membrane protein with six transmembrane helices, TatA and TatB, which share sequence homology, each possess only a short N‐terminal transmembrane helix. Tat translocases in most species are three‐component systems, but in some cases, two‐component TatAC and TatBC systems were described [6, 7, 8].

Integrating the recently determined cryogenic electron microscopy structures of bacterial TatBC systems [9, 10] with a wealth of physiological and mechanistic data, a detailed multi‐step model of how the bacterial Tat system transports folded proteins across biological membranes was recently proposed [11]. The model suggests a dynamic mechanism that proceeds in three distinct phases. In the first phase, the twin‐arginine signal peptide binds specifically within a hydrophobic pocket of the TatBC core complex. This substrate binding triggers the second phase, which involves the recruitment and assembly of TatA oligomers that locally destabilize and thin the lipid bilayer. In the final phase, driven by the proton motive force, the folded cargo protein translocates through this transiently disrupted membrane zone, after which the complex disassembles to reset the system.

Early evidence of the Tat transport pathway came from investigations in plants [12]. In chloroplasts, it is necessary for the integration of photosynthetic proteins into the thylakoid membrane. Mutant plants that lack either plastid TatA, TatB, or TatC are characterized by an albino phenotype [13, 14].

The function of the plant mitochondrial TatC protein (mTatC) remained unclear for a long time. In fact, it was impossible to detect the protein by any method, leading to speculation that it represents a pseudogene. It later was discovered that plant mitochondria include TatB (mTatB), which is nuclear encoded and post translationally imported into the organelles [15, 16]. The presence of a mitochondrial TatBC complex was proposed. Using antibodies against mTatB and mTatC, this complex was later indeed detected in the 1500 kDa range in *Arabidopsis thaliana* (Arabidopsis). Evidence was based on the separation of mitochondrial proteins by blue‐native (BN) polyacrylamide gel electrophoresis (PAGE) followed by immunoblotting [15, 17]. The immune signals were weak, suggesting that the two translocase subunits are rare proteins. In another study, Arabidopsis mTatB was detected in the 530 kDa range upon BN‐PAGE [16]. Insertion of the iron–sulfur (FeS) subunit of complex III of the respiratory chain from Arabidopsis was shown to depend on mitochondrial TatBC [15, 17].

Plant complex III consists of a total of 10 different protein subunits; it is a dimeric protein complex with overall 2 × 10 proteins [18]. The molecular mass of Arabidopsis complex III is 500 kDa; the mass of the Arabidopsis FeS subunit is 23 kDa [19]. Two copies of the FeS subunit are incorporated into the dimeric protein complex at the very end of its assembly process [20] in a step involving TatBC. A TatA protein, which likely is not present in plant mitochondria [13], seems not to be required for this step.

However, it remains a mystery why the plant mTatC protein is so difficult to detect, which up to now has made its detailed characterization very difficult. The mitochondrial proteome of Arabidopsis has been intensively characterized by mass spectrometry for 25 years [16, 21, 22, 23, 24, 26] (and several further studies), but no peptide of mitochondrial TatC has ever been found. For Arabidopsis, meanwhile, more than 1300 different proteins are assigned to mitochondria [26]. Furthermore, no peptide of the mitochondrial TatC protein has ever been detected in any comprehensive analysis of the total proteome of Arabidopsis organs or tissues [27, 28]. In proteome databases for Arabidopsis proteins, such as the SUBcellular location database for Arabidopsis proteins (SUBA) [29] and the Arabidopsis Peptide Atlas Database [30], peptides of mitochondrial TatC are absent.

We here report on a new effort to characterize mTatC using mass spectrometry. To this end, we use sensitive trapped‐ion mobility spectrometry (TIMS) time‐of‐flight (TOF) mass spectrometry (MS) for analyzing mitochondrial fractions of Arabidopsis that have been prefractionated using a complexome profiling approach. We were able to detect various peptides of mTatB and mTatC. The abundance profiles of mTatB and mTatC along a blue‐native polyacrylamide gel lane reveal that they form a protein complex in the 450–530 kDa range. We suggest that TatBC binds to complex III during its late assembly phase for FeS subunit insertion.

## Materials and methods

### Plant material

*Arabidopsis thaliana* Columbia‐0 (Col‐0) heterotrophic cell cultures were established according to [31]. Briefly, Arabidopsis seeds were surface‐sterilized by sequential incubation in 70% ethanol and 6% sodium hypochlorite for 1 min each, followed by three washes with sterile water. The seeds were then plated onto agar plates containing 2.15 g/L Murashige and Skoog basal salt mixture, 0.5 g·L^−1^ MES, 10 g·L^−1^ sucrose, and 1 mL·L^−1^ Gamborg's vitamin solution. For stratification, plates were incubated in the dark at 4 °C for 3 days. Afterwards, the plates were transferred to long day conditions (16 h light, 8 h dark, 22 °C, 120 μmol·sec^−1^·m^−2^ light, 55% humidity). After 10 days, seedlings were dissected into small pieces and the explants were placed on agar plates containing B5 medium to induce callus formation. After 4 weeks of incubation in the dark, calli were transferred to liquid B5 medium and incubated at 22 °C and 100 rpm in the dark. The cells were subcultured weekly into fresh medium.

### Isolation of mitochondria

For isolation of mitochondria, about 100 g of Arabidopsis cells were filtered through two layers of gauze and transferred to 200 mL of chilled disruption buffer [450 mm sucrose, 15 mm MOPS [3‐(N‐Morpholino)‐propane sulphonic acid], 1.5 mM EGTA, 0.6% [w/v] polyvinylpyrrolidone 40, 0.2% [w/v] bovine serum albumin (BSA), 10 mm sodium ascorbate, 10 mm cysteine, 0.2 mm phenylmethylsulfonyl fluoride (PMSF), pH 7.4 [KOH], 4 °C]. Disruption of cells was achieved by homogenizing three times for 15 s with 30 s pauses in a blender. Initial enrichment of mitochondria was achieved by three centrifugation steps: two steps at 2700 × ***g*** and one step at 8300 × ***g*** for 5 min and 4 °C (mitochondria in supernatant). Afterwards, mitochondria were pelleted at 17 000 × ***g*** for 10 min and 4 °C. The pellet was carefully resuspended in ~3 mL cold washing buffer (300 mm sucrose, 10 mm MOPS, 1 mm EGTA, and 0.2 mm PMSF, pH 7.4) and the mitochondria were additionally homogenized with two strokes in a precision homogenisator. The homogenate was then carefully transferred onto three‐step Percoll gradients (10 mL 18% [v/v], 10 mL 23% [v/v] and 10 mL 40% [v/v] Percoll in 300 mm sucrose, 50 mm MOPS, pH 7.4) and centrifuged for 90 min at 70 000 × ***g*** and 4 °C. Finally, the mitochondria were collected at the interphase between the 23% and the 40% phase and washed three times through centrifugation in resuspension buffer (400 mm mannitol, 1 mm EGTA, 10 mm Tricine, and 0.2 mm PMSF, pH 7.4) for 10 min at 14 000 × ***g*** and 4 °C. The purity of our mitochondrial fractions is routinely tested by shotgun proteome analyses (see below) as described before [26, 32]. In short, all identified proteins are assigned to subcellular compartments based on the SUBA database [29]; subsequently, protein abundance values are summed up for all proteins for all compartments. The resulting numbers allow to estimate organelle enrichment. The purity of our mitochondrial fractions from Arabidopsis thaliana is in the range of 80% [26, 32, 33].

### Blue‐native PAGE

Freshly isolated mitochondria were resuspended in resuspension buffer (400 mm mannitol, 1 mm EGTA, 10 mm tricine, 0.2 mm PMSF, pH 7.4) and mitochondrial proteins were quantified via Bradford assay. The concentration was adjusted to 2.5 μg μl^−1^ and 100 μL of protein solution was mixed with 100 μL of digitonin solubilization buffer (30 mm HEPES, 150 mm potassium acetate, 10% [v/v] glycerol, 5% [w/v] digitonin; [24]) and incubated for 20 min on ice. Samples were centrifuged to pellet cellular debris and membrane fragments. A Coomassie solution (750 mm ACA, 5% [w/v] Coomassie brilliant blue G 250) was added to the samples (5% [v/v]). Blue‐native (BN) PAGE was carried out as described previously [34]. We used the Protean II xi cell 2‐D Electrophoresis system (Bio‐Rad, Hercules, USA). The gradient gel used for sample separation was 4.5% to 16% polyacrylamide and separation of proteins took place first, for 45 min at 7 mA and max. 100 V, and afterwards, for 13 h, at 15 mA and max. 500 V. The resulting gels were stained in a Coomassie Brilliant Blue staining solution [1.96% [w/v] ortho‐Phosphoric acid (85%), 9.8% [w/v] ammonium sulfate, 0.1% Coomassie Brilliant Blue G‐250] with 20% [v/v] ethanol.

### Complexome profiling

For complexome profiling BN gel lanes were cut into 40 fractions of 4 mm each (from bottom to top) for data‐dependent acquisition Parallel Accumulation Serial Fragmentation (dda‐PASEF) analysis, and into 30 fractions of 2 mm each for data‐independent acquisition Parallel Accumulation Serial Fragmentation (dia‐PASEF) analysis. In the dia‐PASEF experiment, only the middle region of the gel was subjected to fractionation. Each gel fraction was dehydrated with 100% ACN, and for cysteine alkylation, gel pieces were first incubated with 20 mM DTT for 30 min at 56 °C, followed by 55 mm iodoacetamide at 22 °C for 30 min in the dark. After dehydration with ACN, gel pieces were incubated in 0.1 M ammonium bicarbonate. Trypsin (2 mg·mL^−1^; V5117, sequencing grade, modified, Promega, Madison, Wisconsin, USA) was added and digestion was carried out for 18 h at 37 °C. Peptides were initially extracted by incubating gel pieces in 5% formic acid (FA) and 50% ACN followed by incubation in 100% ACN for 20 min at 37 °C with shaking at 800 × ***g***. The extracted peptides were dried by vacuum centrifugation and stored at −20 °C.

### Peptide clean‐up and MS sample preparation

Samples were prepared for LC–MS analysis as described previously [35]. Peptides were purified using Evotip Pure tips (Evosep Biosystems, Odense, Denmark) according to the manufacturer's manual: Tips were loaded with 20 μL of 0.1% FA in 100% ACN and centrifuged for 1 min at 800 × ***g***. The tips were then preconditioned by soaking in 100% isopropanol for 2 min. Equilibration of the tips was achieved by adding 20 μL of 0.1% FA, and centrifugation for 1 min at 800 × ***g***. The equilibration step was repeated. Samples were loaded onto the tips by centrifugation for 1 min at 800 × ***g***. The loaded tips were washed with 20 μL FA and centrifugation for 1 min at 800 × ***g***. For storage, 100 μL of 0.1% FA was added to the tips and centrifuged for 1 min at 100 × ***g***.

For dda‐PASEF analysis, peptides were reconstituted in 60 μL 0.1% [v/v] FA and 20 μL of each fraction were used. The dia‐PASEF complexome was measured two times. Initially, the peptide concentration was determined using the Pierce Peptide Quantification Kit (Thermo Fisher Scientific, Bremen, Germany) following the manufacturer's instructions. To ensure optimal detection of the low‐abundance mTatC peptides, an initial run was performed using a constant load of 200 ng of peptide per fraction. Subsequently, to generate the representative complexome profiles, a second analysis was conducted where the sample amounts were adjusted to reflect the original protein distribution across the gel. For this purpose, 200 ng of the fraction with the highest peptide concentration was used as a reference, and all other fractions were adjusted proportionally.

### LC‐IMS‐MS/MS analysis

All samples were analyzed using an Evosep One LC system (Evosep Biosystems, Odense, Denmark) connected to a timsTOF Pro IMS quadrupole TOF mass spectrometer (Bruker Daltonics, Bremen, Germany).

For the dda‐PASEF analysis, peptides were separated using a 30 samples‐per‐day (30 SPD) standardized gradient (44 min) on a EV1137 column (Evosep Biosystems, Odense, Denmark). MS parameters were applied as described previously [35]. In short, peptides were ionized via electrospray ionization at a temperature of 180 °C and a capillary voltage of 1600 V. The MS and MS/MS scan ranges were set to 100–1700 m/z with an ion mobility range (expressed as 1/*K*_0_) of 0.85–1.3 V*s/cm^2^. A polygon filtering was applied in the m/z and ion mobility area to exclude the low m/z of singly charged ions for PASEF precursor selection. Ramp and accumulation time were set to 100 ms. The number of PASEF ramps was set to 4 with a ramp rate of 9.42 Hz and a charge minimum of 0, and a maximum of 5. The quadrupole isolation width was set to 2 for m/z = 700 and 3 for m/z = 800. Collision energy was 27 eV for ion mobility (1/*K*_0_) 0.85 V*s/cm^2^ and 45 eV for ion mobility (1/*K*_0_) 1.3 V*s/cm^2^, respectively. Active precursor exclusion was activated with a window of 0.40 min.

dia‐PASEF samples were separated using a 40 samples‐per‐day (40 SPD) Whisper‐zoom standardized gradient (32.5 min) on an Aurora Elite 15 × 75 column (IonOpticks, Fitzroy, Australia). Eluting peptides were analyzed using a timsTOF Pro IMS quadrupole TOF mass spectrometer (Bruker Daltonics, Bremen, Germany) using a custom 16 × 2 window acquisition method from m/z 100 to 1700, and from 1/*K*_0_ = 0.65–1.45 [36]. The collision energy was ramped linearly as a function of the mobility from 59 eV at 1/*K*_0_ = 1.6 Vs cm^−2^ to 20 eV at 1/*K*_0_ = 0.6 Vs cm^−2^.

### Data analysis

A custom database combining entries from TAIR10 and Araport11 and permuted sequences of all the mitochondrial‐encoded genes allowing detection of proteoforms specific to editing of mTatC was used [26].

For the generation of mirror plots and complexome profiles, the dda‐PASEF MS data were analyzed with FragPipe (version 24) [37, 38] using most of the settings described previously [35]. Precursor and fragment mass tolerances were set to 20 ppm, and the ‘mass calibration and parameter optimization’ option was enabled. Protease specificity was configured for trypsin digestion (K and R). Up to two missed cleavage sites were allowed. Variable modifications were set to include methionine oxidation and protein N‐terminal acetylation. Carbamidomethyl cysteine was set as a fixed modification. The allowed peptide lengths were 7–50 amino acid residues and the mass range was set to 500–5000 Da. Peptide‐to‐spectrum matches (PSMs) were rescored by MSBooster (version 1.4.14) [39] and percolator (version 3.7.1) [40] using predictions of peptide retention time, ion mobility, and MS2 spectra provided by KOINA, with the optimal models being chosen automatically [41]. Based on PSMs, identified proteins were inferred by ProteinProphet [42]. Philosopher (version 5.1.3) was used to perform 1% false discovery rate (FDR) filtering at the PSM, ion, peptide, and protein level. Quantification of proteins was performed via IonQuant (version 1.11.20) [43]. The ‘Match‐between‐runs (MBR)’ feature was enabled with a MBR ion FDR of 1%.

Both dia‐PASEF datasets were first analyzed with FragPipe (version 24) [44]. DiaTracer (v2.2.1) was used to extract pseudo‐DDA spectra from the timsTOF dia‐PASEF data. For initial peptide identification and spectral library generation, a library‐free search strategy was employed. These extracted spectra were searched using MSFragger (v4.4.1). Search parameters included ‘stricttrypsin’ digestion with up to two missed cleavages. Precursor and fragment mass tolerances were set to 20 ppm and 15 ppm, respectively. Carbamidomethylation of cysteine was set as a fixed modification, while methionine oxidation and N‐terminal acetylation were allowed as variable modifications. To enhance identification confidence, MSBooster (v1.4.14) utilized deep learning models retrieved via the KOINA service [41]. FragPipe automatically selected the optimal models for the specific dataset through a consensus approach. Peptide‐spectrum matches (PSMs) were validated with Percolator (v3.7.1), and a FDR of 1% was applied at the precursor and protein levels using Philosopher (v5.1.3). The resulting consensus spectral library was subsequently used for protein quantification in DIA‐NN (v1.8.2 beta 8).

Subsequently, all dia‐PASEF data were also analyzed via DIA‐NN (version 2.3) [45]. The analysis was performed against a spectral library (6 142 468 precursors) predicted from the database mentioned above but without the permuted sequences to reduce the search space. Search settings included a fixed mass accuracy of 15 ppm for both MS1 and MS2 and a scan window radius of 6 as recommended by DIA‐NN. Peptides with lengths of 7 to 30 amino acids and typical charge states of 2 to 5 were considered. Peptidoform scoring was enabled, carbamidomethylation of cysteine was set as a fixed modification, and variable modifications (methionine oxidation and N‐terminal methionine excision) were localized with a confidence threshold of > 90%. Match‐between‐runs (MBR) was utilized to minimize missing values across the gel fractions. The final output was filtered at a 1% global q‐value at both the precursor and protein group levels.

## Results

### Determination of theoretically detectable tryptic peptides of Arabidopsis mTatC

The plant mitochondrial TatC protein (mTatC, ATMG00570) has thus far eluded mass spectrometric detection. To systematically evaluate which mTatC peptides are theoretically detectable, we used an Arabidopsis TAIR10/Araport11 database [26], which takes particular consideration of the amino acid positions altered by editing of mitochondrial transcripts. Previous studies identified up to 44 editing sites in *Arabidopsis thaliana* orfx transcripts [46], 32 of which cause amino acid substitutions (Fig. S1). Based on the reported editing frequencies, we prioritized 23 ‘major’ sites with high editing efficiency, as amino acid substitutions at these positions are typically fully reflected in the mature protein. In contrast, editing events at low‐frequency ‘minor’ sites were not considered because the amino acids at these positions normally correspond to the original genomic information, which is consistent with the observation that the detectable mitochondrial proteome predominantly includes proteotypes encoded by transcripts altered at the highly edited transcript positions [26, 32, 47]. The predicted Arabidopsis mTatC comprises 286 amino acids and has a molecular mass of 33.19 kDa.

**Fig. 1:**
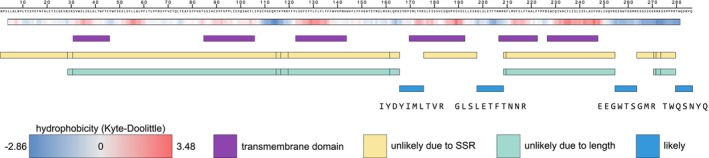
Predicted detectability of tryptic peptides of the Arabidopsis mitochondrial TatC protein (mTatC) protein by mass spectrometry. *Line on the top*: amino acid sequence of the mTatC protein (286 amino acids) as derived from the fully edited mtatc‐mRNA considering all high‐frequency edits (see Fig. 1 for mtatc‐mRNA editing positions). Bar below the amino acid sequence indicates hydrophobicity according to Kyte and Doolittle [48]. Red indicates high hydrophobicity, while blue indicates low hydrophobicity; see color bar at the bottom of the figure to the right. *Purple bars*: Predicted transmembrane (TM) domains. *Yellow bars*: peptides considered unlikely to be observed based on sequence‐specific retention (SSR) calculation (data derived from the Arabidopsis peptide atlas [30]). *Green bars*: peptides that are difficult to detect due to size (smaller than 7 or larger than 30 amino acids). Blue bars: tryptic peptides potentially detectable by MS. Their sequences are given below. Hydrophobicity profiles were calculated via ProtScale using default parameters [49]. Transmembrane domains were predicted via DeepTMHMM version 1.0.57 [50].

Using the curated mTatC sequence, we defined all potential tryptic peptides of Arabidopsis mTatC (assuming zero missed cleavages) and documented their physicochemical properties (Table S1). A systematic evaluation of these peptides revealed that the vast majority are unsuitable for standard LC–MS/MS detection. As shown in Fig. 1, most peptides are either unlikely to be detected due to their extreme sequence‐specific retention (SSR) characteristics—evaluated via the Sequence‐Specific Retention Calculator (SSRCalc), which predicts a peptide's chromatographic elution behavior based on amino acid composition [51]—and high local hydrophobicity (indicated by yellow bars), or fall outside the optimal length range of 7–30 amino acids required for reliable identification (green bars).

Only four peptides emerged as likely candidates for successful mass spectrometric detection: IYDYIMLTVR, GLSLETFTNNR, EEGWTSGMR, and the C‐terminal TWQSNYQ. In addition to their localization in the more accessible, less hydrophobic loop regions or at the protein's C terminus (Fig. 1, blue bars), these four sequences exhibit optimal lengths for LC–MS analysis (ranging from 7 to 11 amino acids). This combination of topological accessibility and favorable chromatographic and physicochemical properties suggests they represent primary targets for the mass‐spectrometry‐based validation of the mTatC protein.

### Mass spectrometric detection and validation of mTatC peptides

Beyond the inherent mass spectrometric challenges posed by its primary structure, mTatC is characterized by exceptionally low transcript abundance and ribosome density [52], likely further complicating its identification even in enriched mitochondrial proteome samples [25, 26]. While strategies such as targeted protein enrichment or extensive peptide‐level fractionation can enhance the detectability of low‐abundance proteins, we opted for a strategy that simultaneously may provide information on protein:protein interactions. To this end, we employed a combination of mitochondrial preparation, classical blue‐native PAGE, and complexome profiling. We reasoned that fractionating mitochondrial proteins and protein complexes in their native state would significantly reduce sample complexity. To maximize sensitivity for protein detection, we utilized timsTOF mass spectrometry. Furthermore, both data‐dependent Parallel Accumulation Serial Fragmentation (dda‐PASEF) as well as data‐independent PASEF (dia‐PASEF) acquisition modes were employed to ensure deep proteome coverage and confident peptide identification.

**Fig. 2:**
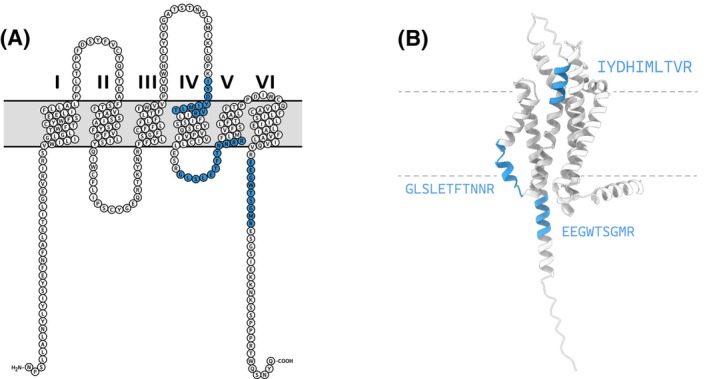
Predicted topology of the Arabidopsis mitochondrial TatC protein (mTatC) protein, positions of identified peptides and their amino acid sequences. (A) 2D topology map of the Arabidopsis mTatC protein generated with Protter [53], highlighting identified peptides (blue). The six predicted transmembrane domains are labeled from I to VI. (B) 3D structural model of the Arabidopsis mTatC protein generated by AlphaFold [54]. Peptides identified by MS are given in blue. Structure visualized in ChimeraX [55].

Using the strategy described, all three internal peptides of the Arabidopsis mTatC protein were successfully identified. While the dda‐PASEF analysis allowed identification of a single peptide (GLSLETFTNNR), the dia‐PASEF data covered three of the four potential candidates: IYDYIMLTVR, GLSLETFTNNR, and EEGWTSGMR (including its methionine‐oxidized variant) (Fig. 2 and Table 1). The C‐terminal peptide, TWQSNYQ, was not detected in the MS/MS datasets. This is likely attributable to its predicted single charge state (*z* = 1), which was excluded from the timsTOF PASEF precursor selection to prioritize multiply charged ions for fragmentation. However, biological evidence indicates that translation terminates prematurely relative to the genomic annotation. In Arabidopsis mitochondria, the annotated stop codon is known to be removed post‐transcriptionally during mRNA 3′ end maturation [56]. Furthermore, ribosome profiling tracks reveal a sharp drop in footprint density upstream of this region, indicating that ribosomes exit the transcript before reaching the sequence encoding the TWQSNYQ peptide [52]. Our inability to detect this peptide lends further support to these findings, suggesting that the synthesized protein likely terminates prior to the genomically predicted stop codon. No additional mTatC peptides beyond those predicted by our theoretical considerations (Fig. 1) could be identified.

**Table 1:** Properties of identified tryptic peptides of the Arabidopsis mitochondrial TatC protein (mTatC) protein. Peptide sequences are given together with flanking amino acids, sequence positions, peptide length and observed modification (Met[ox]). Theoretical m/z represents monoisotopic mass‐to‐charge ratios. Hydrophobicity Index (HR) denotes the dimensionless SSR‐calculated value, where higher values correlate with increased retention time (RT). Note that theoretical HR values were predicted for the native sequences and do not account for the hydrophilic shift induced by methionine oxidation. Experimental Ion Mobility (1/*K*_0_) and average RT were determined via dia‐PASEF acquisition using a 40 SPD Whisper‐Zoom method.

Sequence	aa before	aa after	Start	End	Length	Modification	Theoretical m/z	HR value	Ion mobility (1/*K*_0_) > [V·s·cm^−2^]	Average retention time [min]
EEGWTSGMR	R	E	255	263	9	‐	526.7269^+2^	19.32	0.86	15.21
EEGWTSGMR	R	E	255	263	9	Oxidation (M)	534.7244^+2^	19.32	0.85	12.77
IYDYIMLTVR	K	I	166	175	10	‐	643.8443^+2^	36.91	0.96	25.21
GLSLETFTNNR	R	R	198	208	11	‐	626.3200^+2^	24.9	0.94	20.61

**Fig. 3:**
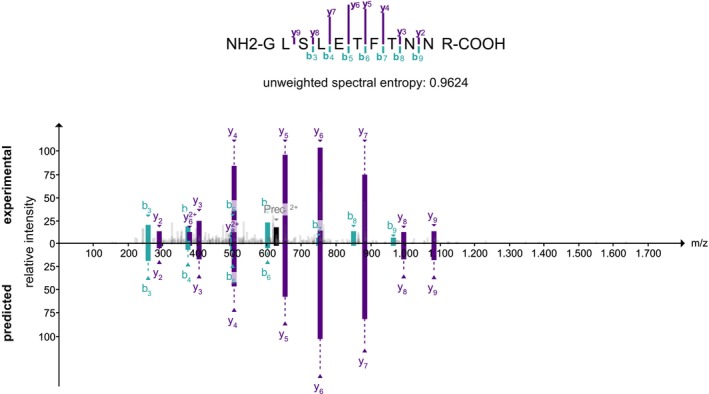
Manual validation of the mitochondrial TatC protein (mTatC) peptide GLSLETFTNN by mirror plot analysis. Comparison of the experimental MS/MS spectrum (top) with the spectrum as predicted by KOINA [41]. Fragments of peptide GLSLETFTNNR are shown in colors (cyan: b‐ions; purple: y‐ions). Nonannotated or alternative peptide peaks in the experimental data set are shown in gray. An unweighted spectral entropy score of 0.9624 indicates a very high degree of similarity between the two plots, strongly supporting the peptide identification. For validation of all other peptides, see Fig. S2.

To ensure the reliability of the automated search engine results, we manually validated the software‐assigned peptides against the raw mass spectrometric data. Figure 3 presents a mirror plot comparing the experimental MS2 spectrum of the peptide identified by FragPipe in dda‐PASEF mode against a theoretical prediction derived from KOINA [41]. The identity of the peptide is clearly confirmed.

To achieve a similarly high level of confidence for the peptides detected by dia‐PASEF, we focused our validation on a consensus set of peptides that were independently identified by two established software packages, FragPipe and DIA‐NN. Similar to the dda‐PASEF data, the identity of these consensus peptides was supported by mirror plots comparing the dia‐MS2 spectra against theoretical predictions (Fig. S2, upper panels), showing very high spectral similarity. Furthermore, the DIA‐NN results were imported into Skyline [57] for a detailed inspection of the extracted ion chromatograms (XICs; Fig. S2, middle and lower panels). The MS1 and MS2 signals demonstrated clean co‐elution and consistent peak shapes, providing an additional layer of evidence for the identification. We conclude that Arabidopsis mTatC has been successfully identified by mass spectrometry.

### Co‐migration analysis of mTatC and mTatB via complexome profiling

**Fig. 4:**
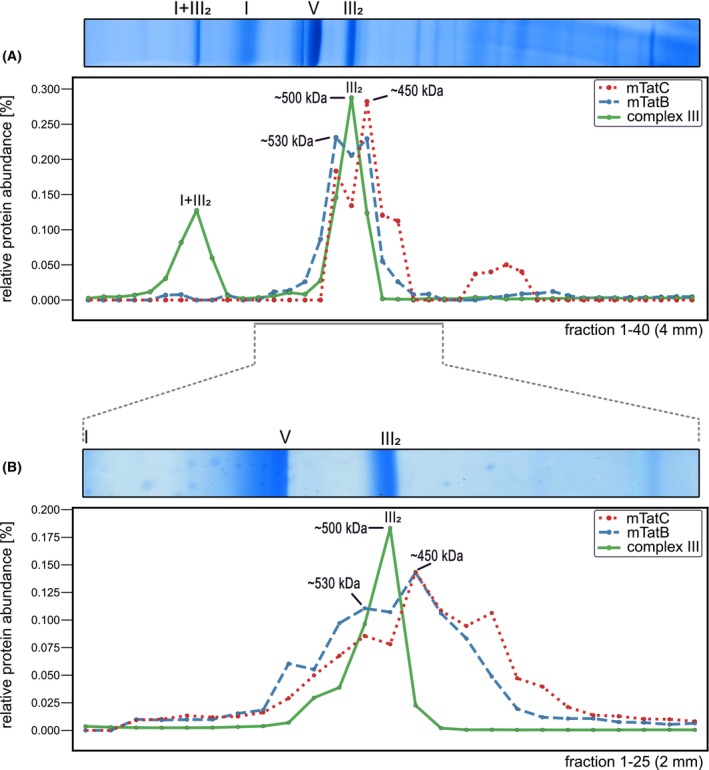
Relative abundance profiles of mitochondrial TatC protein (mTatC) and mTatB compared to subunits of respiratory complex III along blue‐native polyacrylamide gel electrophoresis (BN‐PAGE) gel lanes. Upper panels show blue‐native polyacrylamide gel lanes with separated mitochondrial protein complexes from *Arabidopsis thaliana*, respectively. Lower panels display abundance profiles (normalized intensity) of mTatC, mTatB, and dimeric complex III (average of all 10 subunits) as determined (A) across 40 gel fractions using data‐dependent LC‐IMS‐MS/MS analysis where the mTatC profile is based on a single proteotypic peptide, and (B) across 25 gel fractions using data‐independent LC‐IMS‐MS/MS analysis with the mTatC profile based on four identified peptides. The molecular masses of specific profile peaks are indicated. I: complex I; III_2_: dimeric complex III; V: complex V; I + III_2_: supercomplex formed by complex I and dimeric complex III. Legend: red dotted line, mTatC; blue dashed line, mTatB; green solid line, subunits of complex III. Profiles plotted in Instant Clue [58].

A putative mitochondrial TatB protein (mTatB; At5g43680) has been identified in mitochondrial fractions of Arabidopsis [15, 16]. The protein comprises 232 amino acids and has a molecular mass of 25.03 kDa. Building upon the peptide‐level data for mTatC, we next analyzed our complexome profiling data to compare its abundance profile along BN gels with that of mTatB. The dda‐PASEF data allowed a comprehensive survey across the entire molecular mass range of the BN gel lane, which was dissected into 40 gel fractions (Fig. 4A). Profiles of mTatC and mTatB clearly correlate along the gel lane. Both proteins show peaks at approximately 450 and 530 kDa. Subunits of complex III peak in‐between at about 500 kDa. Minor signals for mTatB or mTatC were detected in the <200 kDa range; furthermore, minor signals were detected for mTatB above 700 kDa.

To obtain data of even higher resolution, complexome profiling was repeated for the 200–1000 kDa range using smaller gel fractions and dia‐PASEF acquisition (Fig. 4B). Consistent with the first complexome, mTatC and mTatB were found to peak at 450 kDa. The second peak at ~530 kDa is visible but less prominent. Another peak at ~350 kDa primarily includes TatC, while a peak at ~600 kDa mainly includes TatB. We conclude that plant mitochondria likely include TatBC complexes of 450 and 530 kDa.

## Discussion

Using a highly sensitive MS approach in combination with complexome profiling, we were able to identify Arabidopsis mTatC. The analysis of mTatC peptides in complexome profiling experiments also allowed us to decipher the native state of the TatC protein. We found that mTatC and mTatB both exhibit abundance peaks at 450 and 530 kDa. We cannot rule out the possibility that the two peaks are in fact a single peak at approximately 500 kDa, which is quenched in a central area by the very high abundance of subunits of mitochondrial complex III. Indeed, the protein amount in the BN gel lanes in the 500 kDa range is significantly higher than the protein amounts in the 450 and 530 kDa ranges (see BN gel lanes in Fig. 4A,B). We find hardly any signal of a TatB and TatC in the 1500 kDa range, which is in contrast to immunological data presented previously [15]. We conclude from our data that Arabidopsis mitochondria likely include mTatBC complexes in the size range of 470–530 kDa.

What could be the nature of these complexes? In bacteria, it has been found that three copies of TatC and three copies of TatB assemble into a hexameric protein complex, which was recently structurally resolved using cryo‐EM [9, 10]. In Arabidopsis, a hexameric protein complex composed of three mTatB and three mTatC subunits would have a molecular mass of about 180 kDa, which has not been observed. To date, there is no clear evidence for the presence of additional Tat subunits in the mitochondria of plants, like TatA [13]. We conclude that the molecular mass of the 470–530 kDa complexes must have another background.

It has been demonstrated that Arabidopsis mTatB and mTatC are involved in the insertion of the FeS subunit into respiratory complex III [15, 17]. In plants, Complex III, which is a functional dimer of 500 kDa, consists of 2 × 10 different subunits [19]. The two FeS subunits are incorporated into dimeric complex III only at the very end of the complex III assembly pathway [20]. We postulate that Arabidopsis mitochondria possibly include heterodimeric mTatBC complexes (approx. 60 kDa) that bind to the final assembly intermediate of complex III (approx. 450 kDa, provided the two FeS subunits are absent). TatB and TatC proteins can be classified as very rare proteins, which explains why it is so challenging to detect them. However, it is likely that only a few copies of these proteins are necessary to catalyze the final steps of complex III assembly. Also, complex III assembly intermediates are present in very small quantities [16, 33].

Further investigations are needed to clarify the composition and function of the 470 to 530 kDa complexes, for example by analyzing orfx deletion mutants. However, methods for altering the mitochondrial genome of plants are still very challenging. Alternatively, mutants with impaired mTatC function could be studied, resulting, for example, from reduced editing rates of mTatC transcripts.

## Author contributions

HPB initiated the project. NR developed the theoretical framework for the project. MD and NR performed all experiments and data analyses, supported by JDJ. MD and NR wrote the first draft of the manuscript with contributions from HPB. All authors contributed to manuscript finalization.

## Conflict of interest

The authors declare no conflict of interest.

## Supporting information


**Fig. S1.** Predicted topology of the Arabidopsis mTatC protein, positions of identified peptides and amino acid positions that are altered by mRNA editing.
**Fig. S2.** Manual validation of DIA‐identified peptides for mTatC using spectral matching and elution profile analysis.
**Table S1.** Theoretical and observed tryptic peptides of the Arabidopsis mTatC protein.

## Data Availability

The raw mass spectrometry proteomics data as well as the FragPipe and DIA‐NN analysis outputs have been deposited to the ProteomeXchange Consortium (http://proteomecentral.proteomexchange.org; Deutsch et al., 2023 [59]) via the Mass Spectrometry Interactive Virtual Environment (MassIVE) repository and can be accessed via:
The dda‐PASEF complexome as MassIVE dataset MSV000101287: https://massive.ucsd.edu/ProteoSAFe/dataset.jsp?accession=MSV000101287
The dia‐PASEF complexome (equal loading) as MassIVE dataset MSV000101285:
https://massive.ucsd.edu/ProteoSAFe/dataset.jsp?accession=MSV000101285
The dia‐PASEF complexome as MassIVE dataset MSV000101397:
https://massive.ucsd.edu/ProteoSAFe/dataset.jsp?accession=MSV000101397
