# Supplementary material for: Identification of the plant mitochondrial OrfX protein: A mass spectrometry approach
Source: FEBS Lett. 2026 Jul 20;600(15):2117–28. doi: 10.1002/1873-3468.70417 (PMC13456617; doi:10.1002/1873-3468.70417)
Supplement: Supplementary file 1 [file FEB2-600-2117-s001.pdf]

Table S1: Theoretical and observed tryptic peptides of the Arabidopsis mTatC protein. Peptides identified by MS analysis are given in bold, all other peptides in normal letters. Peptide sequences are given together with flanking amino acids, sequence positions, peptide length expected charge states with respective monoisotopic m/z ratios, and hydrophobicity values (GRAVY score). Carbamidomethylation of cysteine residues was considered as a fixed modification. Peptides are ordered first by length and second by m/z ratio.

Sequence	amino acid before	amino acid after	Protein Start	Protein End	Length	Charge	m/z (monoisotopic)	hydrophobicity
K	K	N	271	271	1	1	147.11	-
TK	R	Y	115	116	2	2	124.5839	-
NK	K	S	272	273	2	2	131.0815	-
IR	R	S	29	30	2	2	144.6051	-
YNR	K	F	117	119	3	2	226.6162	-
LQPK	K	I	162	165	4	2	243.1577	-
SSPPPR	K	T	274	279	6	2	320.6743	-1.817
TWQSNYQ	R	C-term	280	286	7	1	926.4003	-2.029
ESGSIEK	R	K	264	270	7	2	375.1874	-1.2
**EEGWTSGMR**	**R**	**E**	**255**	**263**	**9**	**2**	**526.7269**	**-1.422**
**IYDYIMLTVR**	**K**	**I**	**166**	**175**	**10**	**2**	**643.8443**	**0.76**
**GLSLETFTNNR**	**R**	**R**	**198**	**208**	**11**	**2**	**626.32**	**-0.655**
ILFISSVCSQVPVIVICLLESR	R	G	176	197	21	2	1266.7	1.714
NPSLLALNYLYISYEFNFALETILGEVR	N-term	I	1	28	28	2	1631.8554	0.411
FFYLSGFCFFLFLFLTFFWVVPNVWHFLYFVGATSTNSLMIK	R	L	120	161	41	3	1718.5384	1.326
FFYLSGFCFFLFLFLTFFWVVPNVWHFLYFVGATSTNSLMIK	R	L	120	161	41	2	2577.3039	1.326
FLMVFSLFTAALFTPPDIWCQIVACFLISLIIELAIFVALIVQVR	R	E	210	254	45	2	2599.937	1.916
SVWILIGLGLTWFTCYWFSEELIFLLALPFLTLPFDSYFVCTQLTEAFSTFVATSSIACSYFVFPLISYQIWCFLIPSCYGEQR	R	T	31	114	84	2	5023.9779	0.977

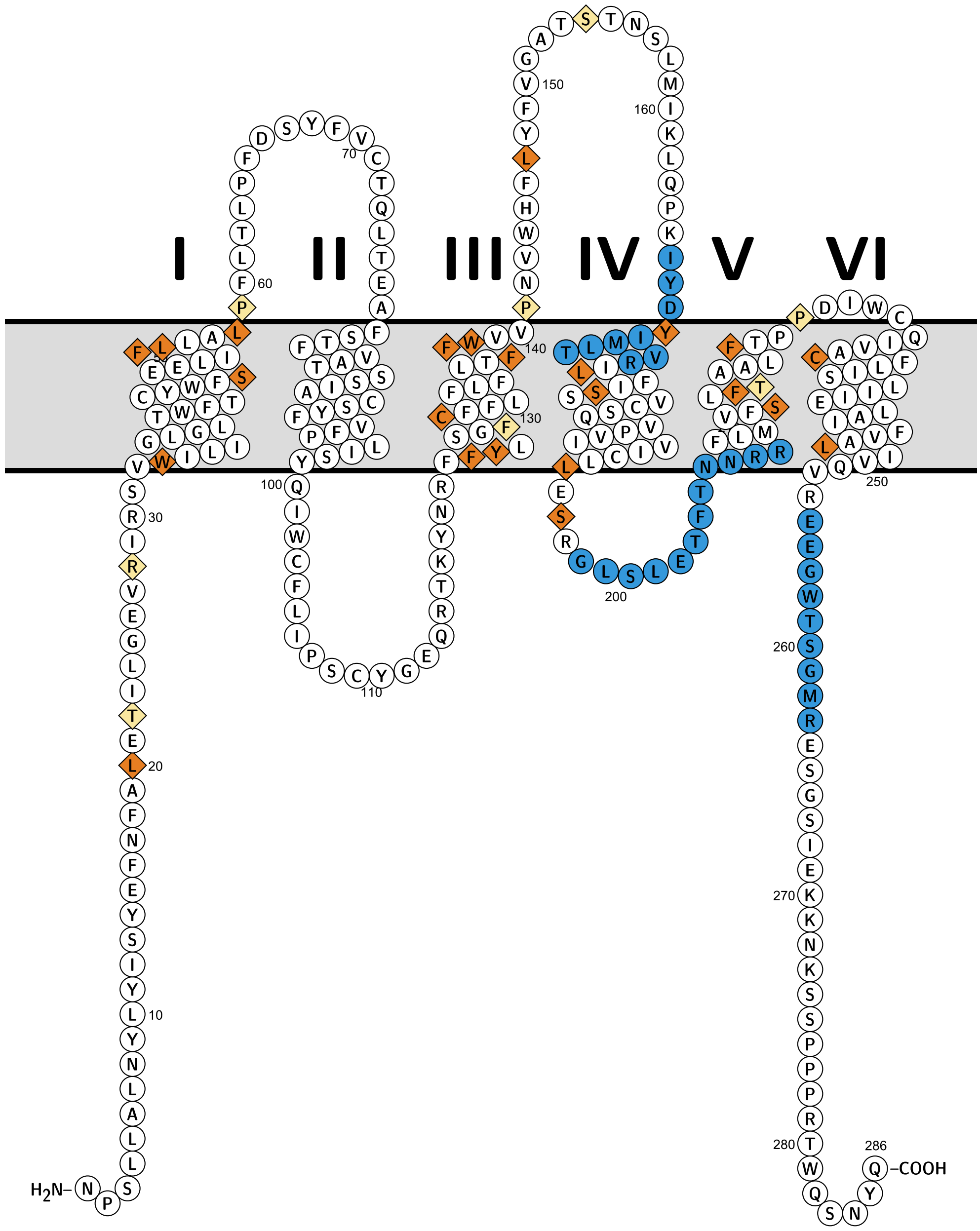

Supplemental Figure S1: Predicted Topology of the Arabidopsis mTatC protein, positions of identified peptides and amino acid positions that are altered by mRNA editing. The membrane topology was visualized using Protter [58], with predicted transmembrane domains labeled I to VI. Experimentally identified peptides are highlighted in blue. Sites of RNA editing, resulting in altered amino acid identities, are indicated by a diamonds and colors, where dark orange denotes high-frequency editing sites, while light orange indicates low-frequency sites. Every 10th amino acid position is labelled by numbers. For our analysis, we only considered high-frequency edits.

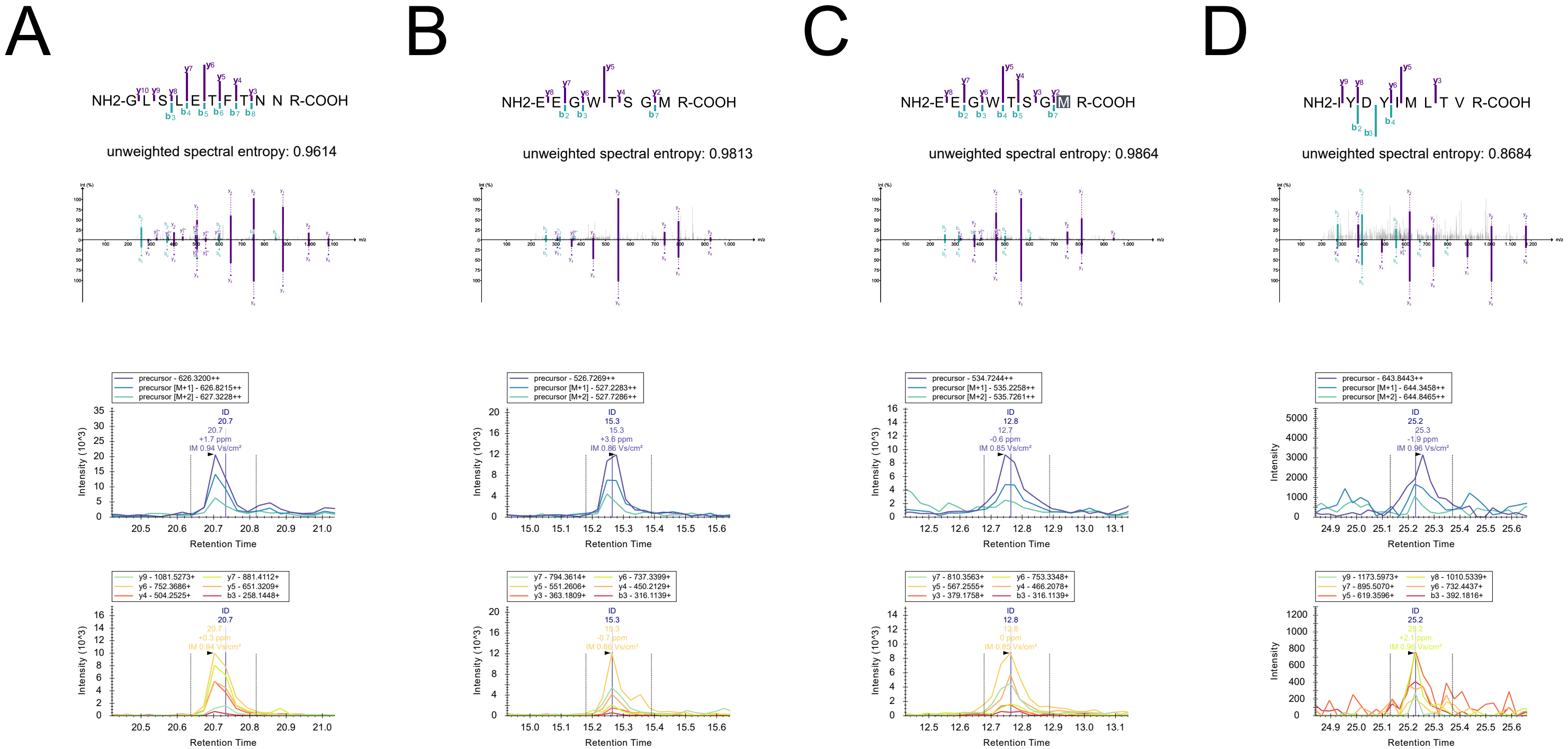

Supplementary Figure S2: Manual validation of DIA-identified peptides for mTatC using spectral matching and elution profile analysis.

Top panel: Mirror plots of annotated experimental MS2 spectra for peptides (A) GLSLETFTNNR, (B) EEGWTSGMR, (C) EEGWTSGMR (including methionine sulfoxide), and (D) IYDYIMLTVR. Experimental data are compared against *in silico* predictions provided by Koina [42]. Purple and teal annotations highlight matching *y*- and *b*-ions, respectively; grey bars represent non-annotated or alternative fragment ions. Middle panel: Extracted ion chromatograms (XICs) of the precursor isotope patterns ([M], [M+1], [M+2]). Labels indicate the precursor *m/z*, charge state (z=2), mass error (ppm), and ion mobility (IM, Vs/cm2) values. Bottom panel: Co-elution profiles of specific fragment ion transitions confirming peptide identity. Vertical dashed lines indicate the integration boundaries used for quantification by DIA-NN. Data were visualized and analyzed using FragPipe-PDV [39] and Skyline [52].
